# Prevalence of Lymph Node Metastasis in Radical Prostatectomy; A Retrospective and Multicenter Study in Iran

**Published:** 2014-03

**Authors:** Mohsen Ayati, Mohammad Reza Nowroozi, Hassan Jamshidian, Elnaz Ayati, Farhad Zarghan

**Affiliations:** Uro-Oncology Research Center (UoRC), Tehran University of Medical Sciences, Tehran, Iran

**Keywords:** Prostate cancer, Prostatectomy, Lymph node dissection

## Abstract

Lymph node (LN) metastasis is considered an important prognostic factor in patients with prostate cancer. The aim of this study was to determine the rate of LN metastasis among an Iranian population who underwent radical prostatectomy (RP) with pelvic LN dissection (PLND).

In a retrospective review of medical records, 450 RP cases were included and the data on LN metastasis were extracted from surgical pathology reports.

Overall, 4.7% of the patients had LN metastasis. The rate of surgical stage T3 (50% vs. 13.5%; P=0.021) and pathological Gleason score ³7 (82.4% vs. 48.8%; P=0.002) was significantly higher among LN-positive patients. All patients with LN metastasis had a serum prostate specific antigen level >4 ng/ml.

The diagnosis of prostate cancer is in an acceptable, but not ideal, stage of the disease; this may be due to screening examinations and tests.

## Introduction

Prostate cancer is the second most commonly diagnosed cancer, after lung cancer, and the sixth leading cause of death due to cancer in men worldwide. It accounts for about 14% of new caners and 6% of cancer-related deaths, based on the global cancer statistics in 2008 (published in 2011).^1^ The most frequently used treatment option for clinically localized adenocarcinoma of the prostate is radical prostatectomy (RP).^2^ Pelvic lymph node dissection (PLND) is recommended to be carried out during this surgical treatment for clinically localized patients with prostate cancer with an elevated risk of lymph node invasion (LNI).^3^^,^^4^ Although there is controversy about the role of PLND for prostate cancer, an important advantage may be to determine the prognosis of patients when LNI is found and it may lead to additional therapeutic opportunities, including adjuvant hormonal therapy after RP.^5^^-^^7^ Lymph node (LN) metastasis is considered an important prognostic factor in patients with prostate cancer. In patients with LNI, it was found that a 10-year cancer-specific survival rate was 47% to 78% in those for whom RP was performed with the immediate hormonal treatment and it was 57% to 62% in those for whom RP was carried out without immediate hormonal therapy.^8^^-^^11^ Daneshmand et al.^11^ in a study on 1936 patients who underwent RP between 1972 and 1999 with PLND found that the rate of LNI was 12.1%. After 1 to 24 years follow-up, the overall median survival was 15 years and the rates of clinical recurrence-free survival at 5, 10, and 15 years were 80%, 65%, and 58%, respectively. The clinical recurrence-free survival rates were significantly correlated with T stage and the number and percentage of positive LNs. The predictive factors and predictive models as well as nomograms for LNI in patients with prostate cancer were investigated in one study, whose results demonstrated that some clinical indicators, including serum prostate-specific antigen (PSA) concentration, clinical stage, and biopsy Gleason score may estimate the risk of LN metastasis.^12^ However, these tools which may be utilized for the purpose of patient selection for PLND usually only provide stratification of a patient’s risk of LNI, with the decision on who should undergo PLND left to the surgeon’s judgment.^13^

The increasing use of PSA testing for the screening and early detection of prostate cancer has led to a dramatic decrease in the rate of LNI to 4-6% in the last decade.^14^ The aim of this study was to determine the rate of LN metastasis among patients with prostate cancer in an Iranian population who underwent RP.

## Patients and Methods

A cross-sectional, observational study with retrospective data obtained from patients’ medical records in a multi-centric study was conducted on clinically localized patients with prostate cancer  who underwent RP between 2006 and 2012 in two large referral hospitals in Tehran, Iran. The study was approved by the institutional Review Board in the Uro-Oncology Research Center (UoRC) related to Tehran University of Medical Sciences. The study protocol was performed in accordance with the Declaration of Helsinki and approved by the Protocol Review Committee of UoRC as well as the Ethics Committee of the University.

To calculate the sample size, N=z2 p (1-p)/d2 formula was used; where N was the minimum required sample size; Z score was 1.96 for the confidence level 95%; p, the proportion affected; and d, the desired precision of this expected proportion. The approximate prevalence of the problem was assumed to be 1.1% (0.011)^15^ and its desired precision (d) was 0.01. Then the minimum sample size was calculated to be 418. The data, including age and pretreatment PSA levels, were obtained from the patients’ medical records. Also, the data on postoperative staging, Gleason score, and LN involvement were extracted from surgical pathology reports.

The results in the categorical variables are given as frequency and percent and in the numerical variables as mean±standard deviation (SD) or standard error of means (SE) as indicated in the legend to the table. Statistical analysis was performed with the Statistical Program for Social Sciences (SPSS 17.0 for Windows; SPSS Inc., Chicago, Illinois, USA). The numerical values were compared between LN-positive and LN-negative patients using the 2-tailed independent *t* test or the Mann-Whitney test where appropriate. A Chi-square or the Fisher exact test was used to compare the categorical variables. In all the tests, a P<0.05 (2-sided) was considered statistically significant.

## Results

Totally, 450 men with prostate cancer were included the study. The mean age of the participants was 66.0±8.6 (range=29-100) years. The mean pretreatment PSA level was 22.3±2.5 ng/ml and most patients (70.5%) had a PSA level between 4.1 and 20 ng/ml (table 1). Of the men, 78.6% were given a pathological stage of T2 and 49.6% had a Gleason score <6.

**Table 1:** Characteristics of the patients who underwent radical prostatectomy and comparison between the patients in whom pelvic lymph node metastasis was observed and those in whom it was not observed

	**Overall** **450 (100%)**	**pN0** **429 (95.3%)**	**pN1** **21 (4.7%)**	**P value**
Age	66.0±8.6	66.0±8.8	64.9±5.4	0.413
PSA level (ng/ml) *	22.3±2.5	22.5±2.7	18.2±3.3	0.380
PSA range (ng/ml)				
0.1-4.0	19 (4.8%)	19 (5.1%)	-	0.398
4.1-10.0	158 (40.2%)	153 (40.8%)	5 (27.8%)	
10.1-20	119 (30.3%)	111 (29.6%)	8 (44.4%)	
>20	97 (24.7%)	92 (24.5%)	5 (27.8%)	
Surgical stage				
T1	8 (4.6%)	8 (4.9%)	-	0.021
T2	136 (78.6%)	131 (80.4%)	5 (50%)	
T3	27 (15.6%)	22 (13.5%)	5 (50%)	
T4	2 (1.2%)	2 (1.2%)	-	
Pathologic Gleason (mean)	6.5±1.2	6.4±1.2	7.5±1.2	0.001
Pathologic Gleason (category)				
≤6	176 (49.6%)	173 (51.2%)	3 (17.6%)	0.002
7	128 (36.1%)	121 (35.8%)	7 (41.2%)	
8-10	51 (14.4%)	44 (13%)	7 (41.2%)	

*mean±SE; PSA: serum prostate-specific antigen

All the patients had undergone a limited LN dissection and a range of 8 to 12 LNs were resected. Overall, 21 (4.7%) of the 450 patients had LN metastasis. The rates of nodal involvement for patients separated by the pretreatment PSA level, surgical staging, and pathological Gleason score are shown in table 1. The rates of the cases with a T3 tumor were 50% and 13.5% in the patients with and without nodal involvement, respectively. Most patients (80.4%) who had no LN metastasis had a T2 tumor (P=0.021). The mean pathologic Gleason score was significantly higher in the LN metastasis group (7.5±1.2 vs. 6.4±1.2; P=0.001). While a Gleason score ≤6 was found in 51.2% of the patients who had no nodal metastasis, it was 7 or more in 82.4% of the positive LN group (0.002). Although all the LN-positive patients had a serum PSA level >4, the mean PSA level was not significantly different between the two groups (P=0.380).

## Discussion

In our study, the rate of LN metastasis was 4.7% in patients who underwent RP and it was associated with pathological staging and Gleason score. The frequency of LNI in our study is in accordance with the previous reports focusing on patients with low-risk prostate cancer. Heidenreich et al.^16^ reported positive LNs in 5.8% of 499 patients who underwent retropubic RP with extended PLND for clinically localized prostate cancer. Even lower rates of LN metastasis were found in a study by Allaf et al.^15^ on 4000 RP surgeries: in 3.2% and 1.1% of patients with extended and limited lymphadenectomy, respectively. These low rates have given rise to debates about the role of PLND as an adjunct of RP in patients with prostate cancer. Although it is currently the most reliable method for LNI diagnosis, recent evidence shows that it is not necessary and is not recommended for low-risk patients with prostate cancer due to the low chance of metastasis. However, it is recommended that at least 10 LNs be dissected for the detection of metastasis and that extended PLND be performed at least for external iliac, obturator, and hypogastric LNs during RP for patients with high or intermediate risk of prostate cancer.^17^

Our study demonstrated that LN-positive patients were associated with higher stage (T3) of the disease and higher Gleason score (7 or more) compared with LN-negative patients. Although a significant association was not observed between LNI and PSA level in the present study, LN metastasis was not found among our patients with prostate cancer with a PSA level ≤4 ng/ml. Similarly, several studies have indicated the association of PSA, clinical Gleason score, and staging with higher risk of LN metastasis.^13^^,^^18^

## Conclusion

The present study demonstrated that the rate of LN metastasis is low (4.7%). The result indicates that the early diagnosis of prostate cancer is in an acceptable, but not ideal, stage of the disease, which may be due to screening examinations and tests. Further studies should be carried out to determine the long-term survival rate of patients with prostate cancer with LN metastasis.
